# Excellent histological results in terms of articular cartilage regeneration after spheroid-based autologous chondrocyte implantation (ACI)

**DOI:** 10.1007/s00167-020-05976-9

**Published:** 2020-04-10

**Authors:** David Grevenstein, Andreas Mamilos, Volker H. Schmitt, Tanja Niedermair, Willi Wagner, C. James Kirkpatrick, Christoph Brochhausen

**Affiliations:** 1 Departement for Orthopaedic Surgery, University Medical Centre of Cologne, Regensburg, Germany; 2 REPAIR-Lab, Institute of Pathology, University of Regensburg, Franz-Josef-Strauß Allee 11, 93053 Regensburg, Germany; 3 Cardiology I, Centre for Cardiology, University Medical Centre, Johannes Gutenberg University of Mainz, Mainz, Germany; 4 Departement for Radiology, University Medical Centre Heidelberg, Heidelberg, Germany; 5 Department of Biomaterials, Sahlgrenska Academy, University of Gothenburg, Gothenborg, Sweden

**Keywords:** Cartilage, Autologous chondrocyte transplantation, Chondrocytes, Spheroids, MACI

## Abstract

**Purpose:**

Traumatic lesions of articular cartilage represent a crucial risk factor for osteoarthritis. Even if several strategies exist to treat such damages, the optimal solution has not yet been found. A new strategy represents the scaffold-free spheroid-based autologous chondrocyte transplantation. In this method, spheroids of chondrocytes are synthesized after chondrocyte isolation and expansion, followed by the implantation in a second intervention.

**Methods:**

Fine Jamshidi-needle biopsies from five patients (one from each patient, Ø 2 mm) treated with a spheroid-based autologous chondrocyte implantation (ACI) after traumatic lesions of the articular cartilage of the knee were analysed histologically and immunohistologically for collagen II, collagen X and aggrecan expression. The indication for a second look arthroscopy was given by arthrofibrosis or meniscus-lesions, respectively. The time between ACI and second-look arthroscopy ranged between 6 and 16 months.

**Results:**

In all patients, the histological examinations revealed an avascular cartilage tissue with a homogenic extracellular matrix. The subchondral bone neither showed bleeding, necrosis nor hypertrophy. A homogenous alcian blue staining indicated high amounts of mucopolysaccharides and glycosaminoglycans. Collagen II staining was highly positive, whereas collagen X staining was negative in every patient, ruling out hypertrophic chondrocyte differentiation. In addition, intense aggrecan staining indicated a strong expression of this extracellular matrix component.

**Conclusion:**

The present case series represents the first histological and immunohistological analyses of spheroid-based ACI in humans. Spheroid-based ACI revealed excellent histological results regarding the regeneration of hyaline articular cartilage. These results indicate that spheroid based ACI is a promising strategy for treating traumatic lesions of the articular cartilage of the knee.

## Introduction

Hyaline cartilage has a unique capacity to answer pressure transformations and is thus critical for the proper function of the musculoskeletal system [11]. However, it has limited regeneration capacity after trauma, leading to degenerative changes of the traumatized cartilage. These changes represent a risk factor for early development of osteoarthritis, resulting in the complete destruction of the joint.

Different strategies have been developed to treat cartilage lesions, such as mosaic arthroplasty, microfracture, autologous matrix-induced chondrogenesis (AMIC) and autologous chondrocyte implantation (ACI) [10, 12]. These strategies were shown to significantly reduce relevant clinical burdens of traumatic cartilage lesions, such as pain and immobility. Moreover, the necessity of total joint replacement could be postponed.

Currently, several Matrix-based ACI (MACI) are in clinical use [1, 9, 18, 19]. However, until today it is not yet clear, which is the optimal one, especially with respect to hyaline cartilage regeneration [4]. Bentley et al. compared ACI with mosaic arthroplasty and revealed excellent clinical, radiological, and histological results [6]. Zeifang et al. compared ACI with MACI and reported no significant differences regarding the IKDC-score, Tegner-activity and the SF-36, but a significantly better result in the Lysholm-score for the ACI-P [20]. A trial comparing periosteal flap covered (ACI-P) and collagen membrane-covered ACI (ACI-C) could not detect histological differences in Safranin-O staining between ACI-P and ACI-C [13, 15]. However, a recent trial reports a high number of failures in osteoarthritic knees and postulate to better profile patients before undergoing the MACI [3].

The spheroid-based ACI represents a relatively new technique. It requires two surgical interventions, one for harvesting chondrocytes and one for the implantation of the generated chondrocyte spheroids [2, 14]. The spheroids are implanted into the defect after debridement and adhere within 20 min at the implantation site. The safety and efficacy of this technique could be demonstrated by clinical studies [5, 17]. However, until today there are no publications regarding the histological outcome after this treatment. In the present case study, we show for the first time histological and immunohistological results of regenerated hyaline cartilage-like tissue after spheroid-based ACT. The present results are of clinical importance as the regenerated cartilage tissue clearly resembles normal hyaline cartilage tissue, which gives relevant implications for its functional properties.

## Materials and methods

Ethical approval for the re-evaluation was given by the Institutional Review Board of the University Regensburg (ID-number: 19-1558-104).

**Fig. 1:**
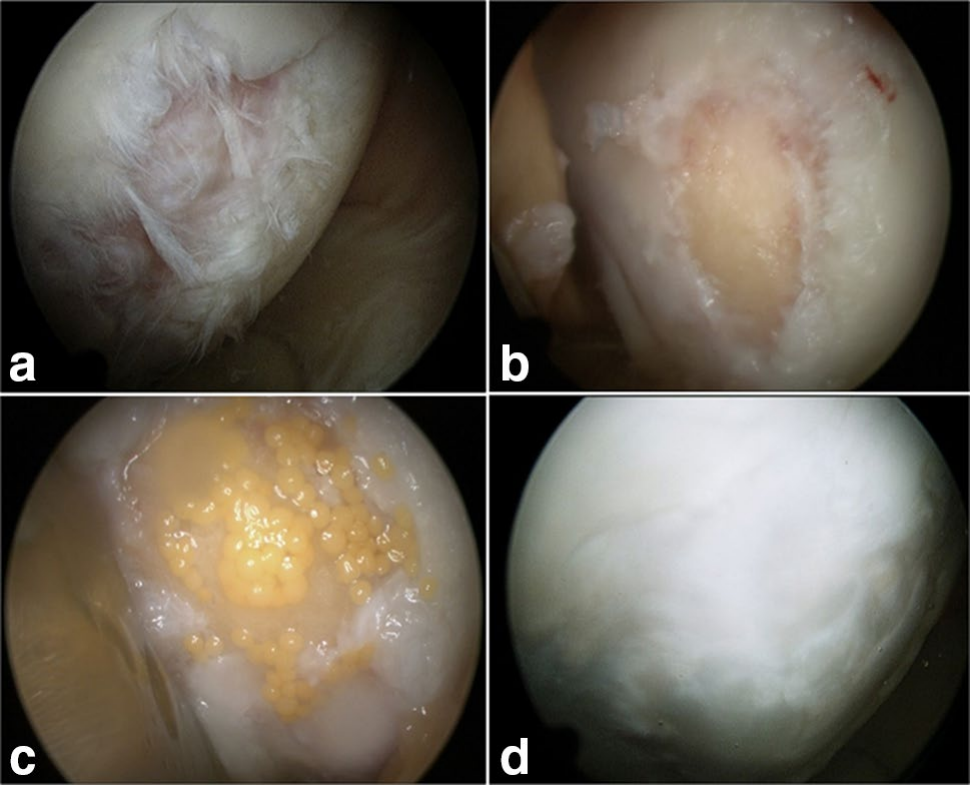
Arthroscopical findings. **a** Traumatic lesion of the articular cartilage; **b** articular cartilage after debridement; **c** Cartilage 20 min after application of the spheroids; **d** regenerated tissue after 6 months

Samples were collected from five patients (one biopsy from each patient) treated with spheroid-based ACI (Codon, Teltow, Germany) due to traumatic lesions followed by re-arthroscopy. All defects were localized on the medial femur condyle. Implantation was performed via mini-arthrotomy (Fig. 1). Re-arthroscopy was necessary on the basis of clinical indications. In three patients a lesion of the medial meniscus, in one patient arthrofibrosis and in one patient persisting pain of unknown origin. Therefore, time-points of re-arthroscopy were not homogenous in the present report. Every patient agreed with a transplant-biopsy in written form to ensure histological analyses of a potential cartilage-hypertrophy versus hypertrophy of the subchondral bone. Biopsies were taken from the central part of the implant using a 2 mm Jamshidi-needle. Specimens were immediately fixed in formalin. The patient characteristics are summarized in Table 1.

**Table 1:** Results of the histological and immunhistological stainings

	Sex F/M	Age [Y]	Time of biopsy [mos]	Defect localization	HE (*N* = 5)	COL II (*N* = 4)	COL X (*N* = 4)	**ACAN** (N = 5)
Range	1/4	37–47	5 ½–16	MFC II (3x) MFC r (2x)	+ + + + +	+ + (Basal) + + (Apikal) + + +	Negative	+ + +
Ø [± SD]		43 [± 4]	11 [± 7]		+ +	+ +	Negative	** + + + **

Biopsies from five patients were collected at different time points after ACI. Staining intensity of haematoxylin (HE), Collagen II (COL II), Collagen X (COL X) and Aggrecan (ACAN) stained paraffin sections was assessed (negative/ +  + / +  + +)

*Y *year, *mos * months, *MFC* medial femur condyle, *SD* standart deviation

### Histological and immunohistological staining

Standardized and automated methods were used in all procedures. The specimens were decalcified, dehydrated and embedded in paraffin (Shandon Pathcentre, Thermo Electron Corporation, USA). Sections of 5 µm thickness were deparaffinized and histological staining was performed with haematoxylin/eosin and alcian blue. Immunohistological detection was performed for collagen II, collagen X and aggrecan (Table 2) in a fully automated system (Histostainer Plus, Dako, Hamburg, Germany).

**Table 2:** Antibodies used for histological and immunohistological stainings

Antigen	Supplier information	Dilution	Blocking buffer/antibody diluent
Collagen II	Nr. II-II6B3, DSHB, Iowa, USA	1:40	TNB buffer
Collagen X	Dako EnVision Flex, HRP, Dab, K8010, Dako, Glostrup, Denmark	1:50	Citrate buffer
Aggrecan	MS MAB Anti-Hu-Aggrecan, Invitrogen, Carlsbad/CA, USA	1:1000	Citrate buffer

### Histological evaluation

Specimens were histologically analysed. The existence of hyaline cartilage-like tissue, fibrous tissue and potential vascularization were examined. Furthermore, the potential hypertrophy of the subchondral bone was described.

### Immunohistological evaluation

The evaluation was performed by two independent double-blinded investigators (D.G. and C.B.) using a visual-based scheme in which the stained area and the intensity of the staining were scored semi-quantitatively using a wide-spread scheme in histopathology. The staining intensity was evaluated as zero (negative/no staining), + (slight positive), +  + (moderate) and +  +  + (strongly positive). A positive control (+ +  + /strongly positive) and a negative control (0/negative) were used as references. No test of significance was possible due to the very low number of patients. Therefore, the focus was placed on the exact description of the tissue. The findings were documented by whole slide imaging using a Digital Microscope and Scanner Device (M8, PreciPoint, Freising, Germany).

## Results

### Histological and histochemical results

Histologically, an avascular cartilage tissue with an intact cartilage-bone border interface was observed in the regenerated tissue in all patients. Cells within the regenerated tissue showed a round and chondrocytic phenotype with cluster-formation. In three patients the typical layer-based architecture with flattened chondrocytes in the apical layer was detectable. The alcian blue staining revealed intensive and homogenous staining of the extracellular matrix in all patients, which represents an indicator for the presence of glycosaminoglycans and mucopolysaccharides. In three of five patients, the staining was intensive in the basal half of the biopsy and decreased in the apical direction, corresponding to the three patients with the typical layer-based structure. The other two patients showed a homogeneous alcian blue staining. There were no signs for degenerative changes, such as demarked collagen fibres in the tissue. Hypertrophy of the subchondral bone was not detected in any of the five analysed patients.

### Immunohistological results

**Fig. 2:**
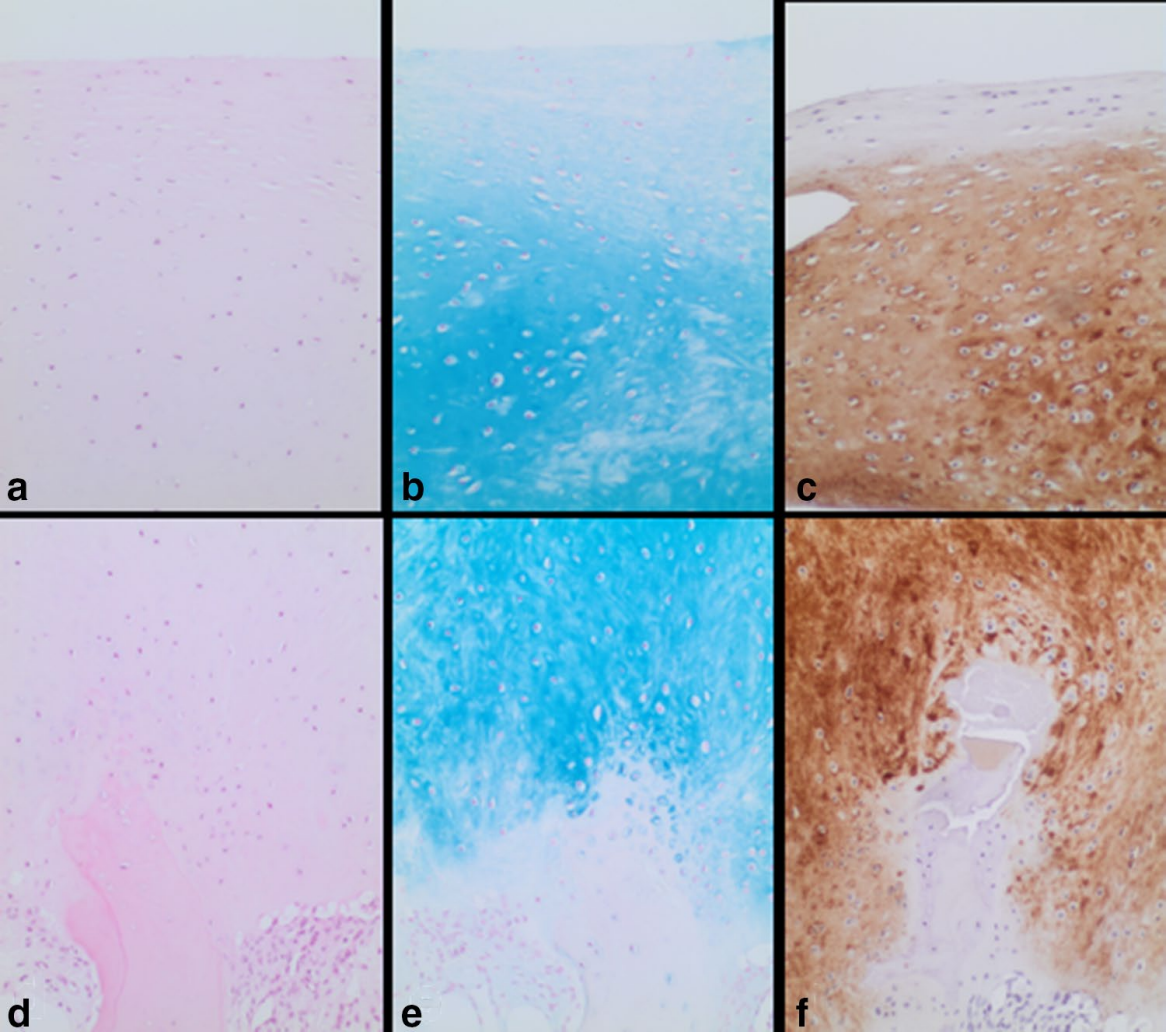
Histological and immunohistological findings. Completely avascular chondroid tissue with a strict cartilage-bone border and round, chondrocytic cells with amorphous extracellular matrix (**a**, **d**). Strong and homogenous alcian blue-positive staining of the extracellular matrix, with a slight decrease of intensity in the apical layers (**b**, **e**). Immunohistological detection of collagen II (**c**, **f**) (magnification × 200)

Immunohistologically, one biopsy showed an intensive and homogenous expression of collagen II within the extracellular matrix. Two biopsies showed an intensive expression of collagen II in the basal and middle zone of the biopsy with a slight decrease of intensity in the apical half of the specimen. In one patient, higher expression in the apical zone could be detected than in the middle and basal zone. One patient was not evaluable due to loss of the cartilage from the biopsy after performing the histological staining. The immunohistological staining for aggrecan revealed a homogenous strong expression in all patients. No spatial differences of the aggrecan positivity could be detected in the basal, middle or apical zone of the biopsies. The immunohistological staining for collagen X antigen was negative in four patients. In one patient no more cartilage tissue was seen in the biopsy. The results of the immunohistological evaluation are summarized in Table 1. The histological and immunohistological findings are documented in Fig. 2.

## Discussion

The present study demonstrates for the first time that the regenerated cartilage tissue after spheroid-based ACI is histologically, histochemically and immunohistochemically very similar to hyaline articular cartilage. The typical layer-based architecture with a deep, middle and apical zone was identified in three patients. In addition, these three patients demonstrated a typical apical zone with two to three layers of flattened chondrocytes and a decrease of alcian blue-positive glycosaminoglycans, which is well known from initial articular cartilage. Specimens with high amounts of glycosaminoglycans, high expression of collagen type II and cells with chondroid phenotype, but without the typical layer-based architecture was defined as “very similar to hyaline articular cartilage”, which was present already 5 ½ months after ACI and almost of similar structure in the specimen which was taken 16 months after ACI. These findings implicate a good regenerative potential of the spheroids, making them an attractive alternative to other methods where changes in the regenerative tissue and long-term failures have been described [3, 6]. Long-term follow-up studies should clarify if spheroid-based ACI also reveals a good long-term performance with respect to proper cartilage regeneration and good long-term preservation of the regenerated tissue. For that purpose, histological analyses are the method of choice, since clinical or radiological examinations were not able to verify the quality of the regenerated tissue [7, 16, 20]. However, further long-term follow-up trials are mandatory for a better understanding of the cellular mechanisms involved in cartilage regeneration and preservation, as well as to give a proper base for the comparison with single-stage techniques as e.g. microfracture, which may be easier to perform [18]. From the clinical point of view, a recent study demonstrated a substantial improvement of various clinical outcomes parameters after 36 months after matrix-associated, spheroid-based ACI compared to microfracturing [14]. Nevertheless, the ACI should also prove, that the regenerated tissue is indeed a noticeable improvement in tissue quality which is mandatory for long-term function.

In this study biopsies taken between 5 ½ and 16 months after ACI clearly demonstrated the regeneration of predominantly hyaline cartilage-tissue. Nevertheless, the limited number of specimens without a homogenous study cohort is a major limitation of the present case-study. Further clinical trials with a larger number of patients have to be performed to consolidate these results and to generate statistically significant data [3]. The indication for the biopsy in this report was given by a potential hypertrophy of the subchondral bone, which had to be excluded by histomorphological analyses.

## Conclusion

The present study represents the first histological and immunohistological analysis of spheroid-based ACI. It shows that spheroid-based ACI yields excellent histological results in terms of regeneration with predominantly hyaline articular cartilage.
